# MiR-320 inhibits the growth of glioma cells through downregulating PBX3

**DOI:** 10.1186/s40659-017-0137-4

**Published:** 2017-09-21

**Authors:** Cuicui Pan, Hua Gao, Ni Zheng, Qi Gao, Yuanquan Si, Yueran Zhao

**Affiliations:** 1 Department of Clinical Laboratory, Shandong Provincial Hospital Affiliated to Shandong University, Jinan, China; 2 Central Laboratory, Provincial Hospital Affiliated to Shandong University, 544 Jingwu Road, Jinan, 250021 China

**Keywords:** Glioma, Luciferase reporter assay, MiR-320, PBX3

## Abstract

**Background:**

MiR-320 is downregulated in multiple cancers, including glioma and acts as tumor suppressor through inhibiting tumor cells proliferation and inducing apoptosis. PBX3 (Pre-B cell leukemia homeobox 3), a putative target gene of miR-320, has been reported to be upregulated in various tumors and promote tumor cell growth through regulating MAKP/ERK pathway. This study aimed to verify whether miR-320 influences glioma cells growth through regulating PBX3.

**Methods:**

Twenty-four human glioma and paired adjacent nontumorous tissues were collected for determination of miR-320 and PBX3 expression using RT-qPCR and western blot assays. Luciferase reporter assay was performed to verify the interaction between miR-320 and its targeting sequence in the 3′ UTR of PBX3 in glioma cells U87 and U251. Increased miR-320 level in U87 and U251 cells was achieved through miR-320 mimic transfection and the effect of which on glioma cells growth, proliferation, cell cycle, apoptosis and activation of Raf-1/MAPK pathway was determined using MTT, colony formation, flow cytometry and western blot assays. PBX3 knockdown was performed using shPBX3 and the influence on MAPK pathway activation was evaluated.

**Results:**

MiR-320 downregulation and PBX3 upregulation was found in glioma tissues. Luciferase reporter assays identified miR-320 directly blinds to the 3′ UTR of PBX3 in glioma cells. MiR-320 mimic transfection suppressed glioma cells proliferation, and induced cell cycle arrest and apoptosis. Both miR-320 overexpression and PBX3 knockdown inhibited Raf-1/MAPK activation.

**Conclusion:**

MiR-320 may suppress glioma cells growth and induced apoptosis through the PBX3/Raf-1/MAPK axis, and miR-320 oligonucleotides may be a potential cancer therapeutic for glioma.

## Background

Glioma is one of the most common forms of neural malignancy and is a highly infiltrating, aggressive brain cancer with no available curative treatment [1]. Despite therapeutic advances, the 5-year survival rate of patients with low-grade gliomas (World Health Organization [WHO] grade I and II) is approximately 30 to 70%, whereas the median survival duration of patients with glioblastoma multiforme (GBM) (grade IV) ranges from 9 to 12 months [2, 3]. Thus, it is quite urgent to investigate the mechanisms underlying the development and progression of glioma in order to identify sensitive and specific early biomarkers for diagnosis and prognosis.

MicroRNAs (miRNAs) are a class of short, endogenous, non-coding RNA molecules that function as post-transcriptional gene regulators through binding to complementary sequences in the 3′UTRs of target mRNA transcripts [4, 5]. Growing evidence has shown that aberrant expression of miRNAs are involved in the progression and development of human cancers, either as oncogenes or tumor suppressors [6, 7]. Previous investigations have indicated that miR-320 is involved in the development of several human tumors [8–12]. Dong et al. found miR-320 showed significantly low expression in glioblastoma patients [13], however, the exact role of miR-320 in glioma occurrence and development remains unknown.

Pre-B-cell leukemia homeobox (PBX) refers to a family of transcription factors, including PBX1, PBX2 and PBX3. PBX3 has been continuously reported to be associated with tumor growth and progression. Li et al. found PBX3 was an important cofactor of HOXA9 in leukemogenesis [14]. HOXA/PBX3 knockdown impaired leukemia growth and sensitized cells to standard chemotherapy [15]. Recently, PBX3 was reported to be upregulated in gastric cancer and to regulate tumor cell proliferation [16]. Han et al. demonstrated PBX3 promoted migration and invasion of colorectal cancer cells via activation of MAPK/ERK signaling pathway [17]. However, no data exist concerning the role of PBX3 in the progression of glioma. In addition, as a putative target gene of miR-320, whether miR-320 functions through regulating PBX3 remains unknown.

In the present research we identified PBX3 was regulated by miR-320 in glioma cells. MiR-320 overexpression suppressed glioma cells proliferation and induced cell cycle arrest and apoptosis. Either miR-320 overexpression or PBX3 knockdown induced inactivation of MAPK pathway.

## Methods

### Ethics statement

This study was approved by the hospital ethics committee, and written informed consent was obtained from all of the patients.

### Clinical specimens

Twenty-four human glioma tissues, including eleven low-grade gliomas (two grade I and eleven grade II tumors) and thirteen high-grade gliomas (five grade III and eleven grade IV tumors), were obtained from the department of neurosurgery of provincial hospital affiliated to Shandong University. The glioma specimens were verified and classified according to the WHO classification of tumors by two experienced clinical pathologists. All tissue samples resected during surgery were immediately frozen in liquid nitrogen for subsequent total RNA extraction.

### Cell culture

U87 and U251 glioma cell lines were purchased from Cell bank of chinese academy of sciences (Shanghai, China). Cells were cultured in Dulbecco’s modified eagle’s medium (DMEM; Sigma Aldrich, St. Louis, MO, USA) supplemented with 10% fetal bovine serum. The cultures were maintained at 37 °C in a humidified atmosphere with 5% CO_2_.

### Transfection of oligonucleotides and plasmid vectors

MiR-320 mimics, DNA template oligonucleotide corresponding to PBX3 and their control oligonucleotides were obtained from Genepharma (Shanghai, China). All of the above sequences were inserted into the BglII and HindIII enzyme sites of pSUPER.retro vector, respectively. The transfection were performed using Lipofectamine™ 2000 (Invitrogen, USA) according to the instructions provided by the manufacturer.

### Luciferase assays

The 3′-UTR of PBX3 was amplified and cloned downstream of firefly luciferase coding region in the pMir-Report vector (pMir-REPORTTM; Ambion Life Technologies). Mutations were introduced into the potential miR-320 binding sites using the QuikChange site-directed mutagenesis kit (Stratagene, Agilent, San Diego, CA, USA). Firefly luciferase reporters, Renilla luciferase pRL-TK vector (used as internal control, Promega, USA) and miR-320 mimics were co-transfected into the U87 and U251 cells. Cells were collected 36 h after transfection and assayed for luciferase activity using the Dual-Luciferase Reporter Assay System (Promega Corporation).

### Quantitative real-time polymerase chain reaction (qRT-PCR)

Total RNAs in tissues and cell lines were extracted by using TRIzol reagent (Invitrogen, USA). For miRNA analysis, mature miRNAs were reverse-transcribed, and real-time PCR was performed using TaqMan microRNA assays (Applied Biosystems). For mRNA analysis, real-time PCR was performed using Power SYBR® green PCR master mix on an ABI 7900HT series PCR machine (Applied Biosystems). Quantization of U6 snRNA and GAPDH was used to normalize the expression level of miRNAs and mRNA respectively. The primer sequences were as follows: miR-320, F, 5′-ACA CTC CAG CTG GGA AAA GCT GGG TTG AGA-3′ and R, 5′-TGG TGT CGT GGA GTC G-3; PBX3, F, 5′-CAA GTC GGA GCC AAT GTG-3′ and R, 5′-ATG TAG CTC AGG GAA AAG TG-3′; GAPDH, F, 5′-TGA AGG TCG GAG TCA ACG GAT TTG GT-3′ and R, 5′-CAT GTG GGC CAT GAG GTC CAC CAC-3′; U6, F, 5′-CGC TTC GGC AGC ACA TAT AC-3′ and R, 5′-TTC ACG AAT TTG CGT GTC AT-3′.

### Cell cycle analysis

U87 and U251 cells were harvested 48 h post-transfection and washed with ice-cold phosphate buffered saline (PBS). After fixed in 70% ethanol for 24 h, the cells were re-suspended in 200 μl of PBS containing 50 μg/mL of propidium iodide (Sigma), 10 μg/mL RNase A, 0.1% sodium citrate and 0.1% Triton X-100, incubated for 1 h at 37 °C in the dark. Cell cycle was analyzed immediately using the flow cytometer (Millipore Guava).

### Cell proliferation assays

The cell proliferation were determined by MTT and colony formation assays. For MTT assay, U87 and U251 cells with a concentration of 2 × 10^3^ cells per well were seeded into 96-well plates for 24 h before transfection. For quantitation of cell viability, 25 μl of MTT stock solution (KeyGEN, China) was added to each well and followed an incubation at 37 °C for 4 h at 1, 2, 3, 4, 5, 6 and 7 days after transfection. Absorbance value of each well was measured spectrophotometrically at 570 nm. For the colony formation assay, cells transfected with miR-320 mimics were placed into 6-well plate and maintained in media containing for 2 weeks. Colonies were fixed with methanol and stained with 0.1% crystal violet (Sigma, St. Louis, Mo). Visible colonies were manually counted.

### Cell apoptosis assays

U87 and U251 cells were plated in six-well plates and transfected with miR-320 mimics. For measurement of cell apoptosis, the cells were harvested 48 h post-transfection and incubated with Annexin V/Propidium Iodide (Sigma-Aldrich). The apoptotic cells were detected and quantified using flow cytometry (Becton–Dickinson) according to the manufacturer’s instructions.

### Western blot assay

Total protein was extracted using RIPA lysis buffer (Cell Signaling, USA). Concentrations of each protein sample were determined by BCA Assay Kit (KeyGEN, China). Equal amounts of proteins in each sample were separated by 10% sodium dodecyl sulfatepolyacrylamide gel electrophoresis (SDS-PAGE) and transferred to polyvinylidene fluoride (PVDF) membranes (Millipore Corporation).

After blocked with a 5% skim milk solution, the blots were incubated with primary antibodies against PBX3 (), caspase-3, Raf-1, p38, ERK1/2, ERK5 and JNK overnight, followed by HRP conjugated secondary antibody (Santa Cruz). Blots were visualized by using an enhanced chemiluminescence detection kit according to the company’s instruction (ECL, Amersham Corp.). Immunoreactive bands were quantified using ImageQuant (Bio-Rad) and normalized to GAPDH.

### Statistical analysis

All experiments were performed at least three times, and all samples were analyzed in triplicates. Data are represented as mean ± SD. Statistical difference between each group was assessed by Student’s t test and ANOVA analysis using SPSS 12.0 software. A *P* < 0.05 was considered to be statistically significant.

## Results

### MiR-320 expression was down-regulated, while PBX3 was up-regulated in glioma tissues

**Fig. 1:**
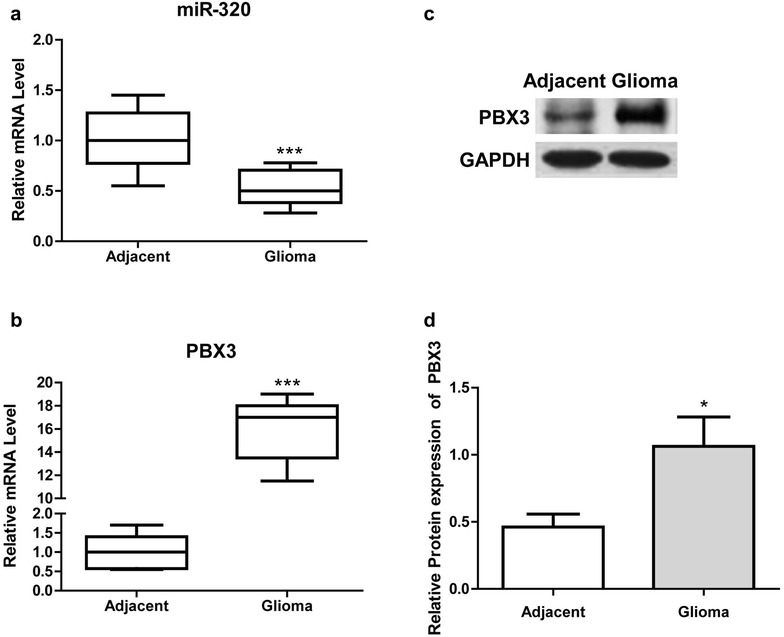
Altered expression of miR-320 and PBX3 in glioma tissues. **a** and **b** Measurement of miR-320 and PBX3 expression in glioma and adjacent tissues of 24 patients using RT-qPCR. **c** A representative result of western blot analysis for PBX3 protein expression in glioma and adjacent tissues. **d**Statistical results of western blot analysis of more than 5 randomly selected paired tissues. * *P* < 0.05, *** *P* < 0.001, compared with adjacent tissues

MiR-320 and PBX3 expression in glioma tissues and adjacent healthy tissues was determined using qRT-PCR. The results showed miR–320 was significantly reduced in glioma tissues in comparison with that of adjacent healthy tissues, while PBX3 expression was detected to be significantly increased in glioma tissues (Fig. 1a, b). Western blot analysis also showed that PBX3 protein expression was significantly increased in glioma tissues compared with adjacent health tissues (Fig. 1c, d).

### miR–320 directly targets PBX3 in U87 and U251 cells

**Fig. 2:**
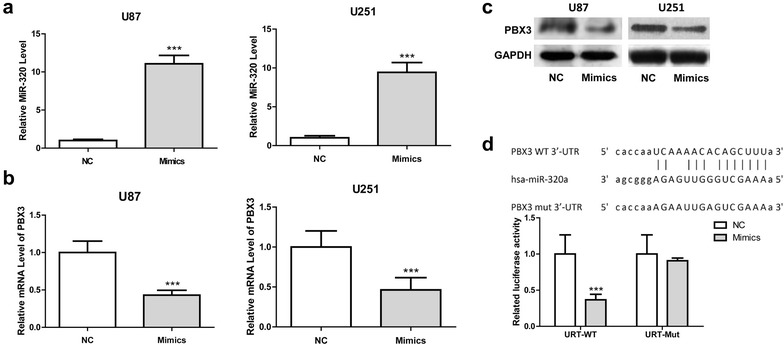
MiR-320 regulates PBX3 expression in glioma cells. **a** miR-320 expression in U87 and U251 cells following transfection with miR-320 mimics or NC, and miR-320 expression was significantly increased in cells transfected with miR-320 mimics in comparison with that with NC. **b** PBX3 expression in U87 and U251 cells following transfection with miR-320 or NC, and PBX3 expression was significantly reduced by miR-320 compared with NC. **c** Western blot of PBX3 in U87 and U251 cells transfected with miR-320 mimics or NC, and protein expression of PBX3 was significantly reduced. **d** Computer prediction of miR-320 binding sites in the 3′UTR of PBX3 gene. Luciferase reporter assays that cells were transfected with 100 ng of wild-type 3′-UTR-reporter or mutant constructs with 100 nM of the miR-320 mimic or NC. ***P < 0.001, compared with negative control

Bioinformatics approaches suggested the gene encoding PBX3 is a putative target gene of miR-320. To determine whether PBX3 expression was regulated by miR-320 in glioma cells, U87 and U251 cells were transfected with miR-320 mimic, and the expression of miR-320 and PBX3 was determined using qRT-PCR and western blot assays. The results showed miR-320 expression was significantly increased by miR-320 mimics, while PBX3 expression was significantly reduced by miR-320 mimics (as shown in Fig. 2a–c). Luciferase reporter assay showed that over-expression of miR-320 led to a marked decrease of Renilla luciferase activity, which was specifically abolished by the mutation of the corresponding anti-seed sequence in 3′ UTR of PBX3 (Fig. 2d). These results suggested that miR-320 directly modulate PBX3 expression by direct binding.

### MiR-320 suppressed glioma cells proliferation through inducing cell cycle arrest at G0/G1 phase

**Fig. 3:**
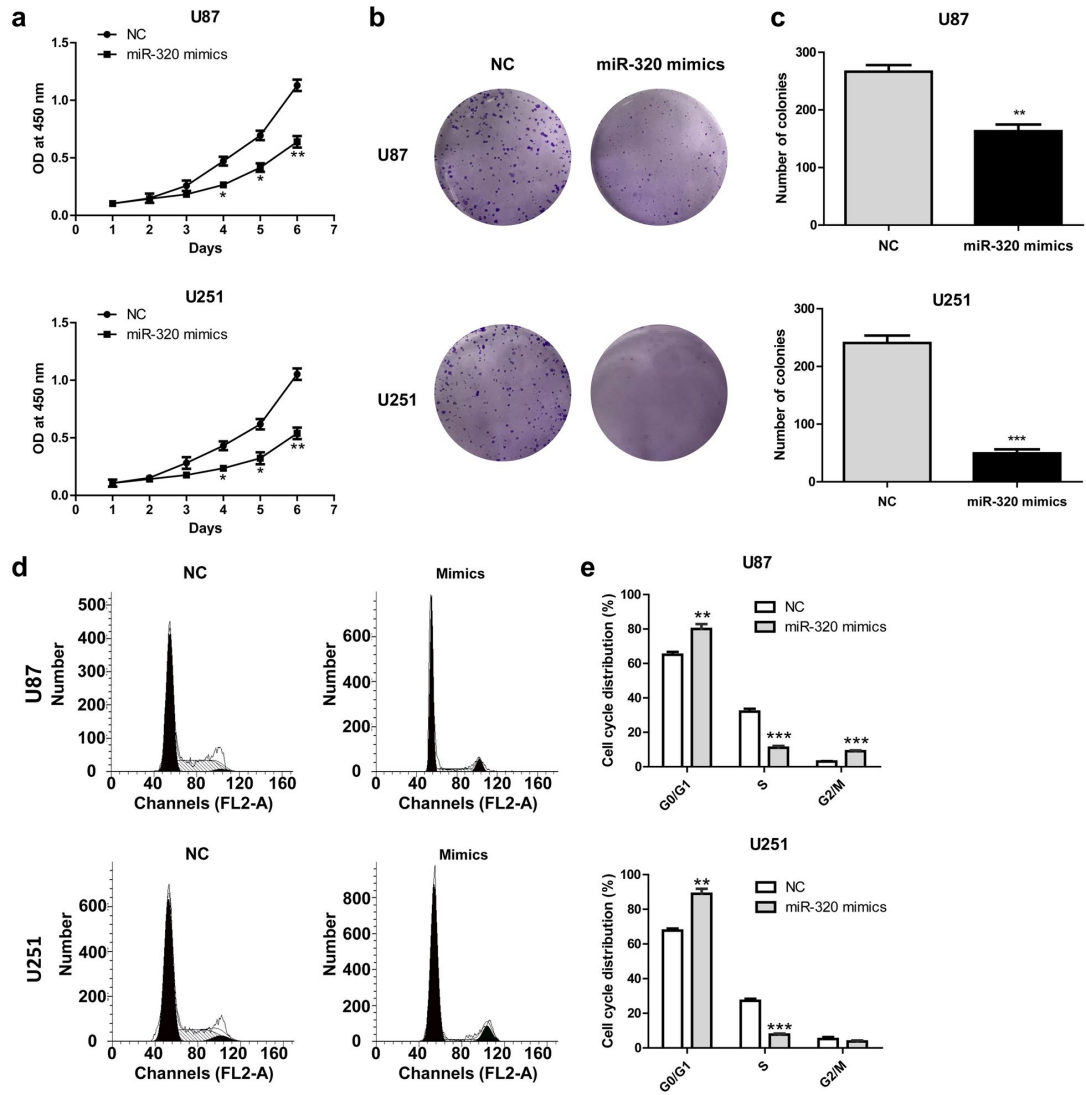
MiR-320 mimics influences the proliferation of U87 and U251. **a** Growth curves of U87 and U251 cells transfected with miR-320 or NC. **b** and **c** U87 and U251 cells transfected with miR-320 mimics were cultured for 2 weeks. Colonies were fixed with methanol and stained with 0.1% crystal violet. Visible colonies were manually counted. **d** Flow cytometric analysis of the indicated glioma cells transfected with NC or miR-320. **e** Cell cycle distribution of U87 and U251 cells transfected with miR-320 mimics or NC. *P < 0.05, **P < 0.01, ***P < 0.001, compared with negative control (at the same time point)

To investigate the biological function of miR-320 in glioma cells, exogenous miR-320 expression by mimic transfection was performed initially. Transfection of mimics significantly suppressed cell proliferation rates and colony formation abilities in both glioma cell lines, U87 and U251 (Fig. 3a–c). We then explored the effect of miR-320 expression on cell cycle progression by flow cytometry methods. Compared to the cells transfected with control mimics, glioma cells with miR-320 transfection showed redistributed cell cycle progression with an increased proportion of cells in G0/G1 arrest (Fig. 3d, e).

### MiR-320 induced apoptosis in glioma cells

**Fig. 4:**
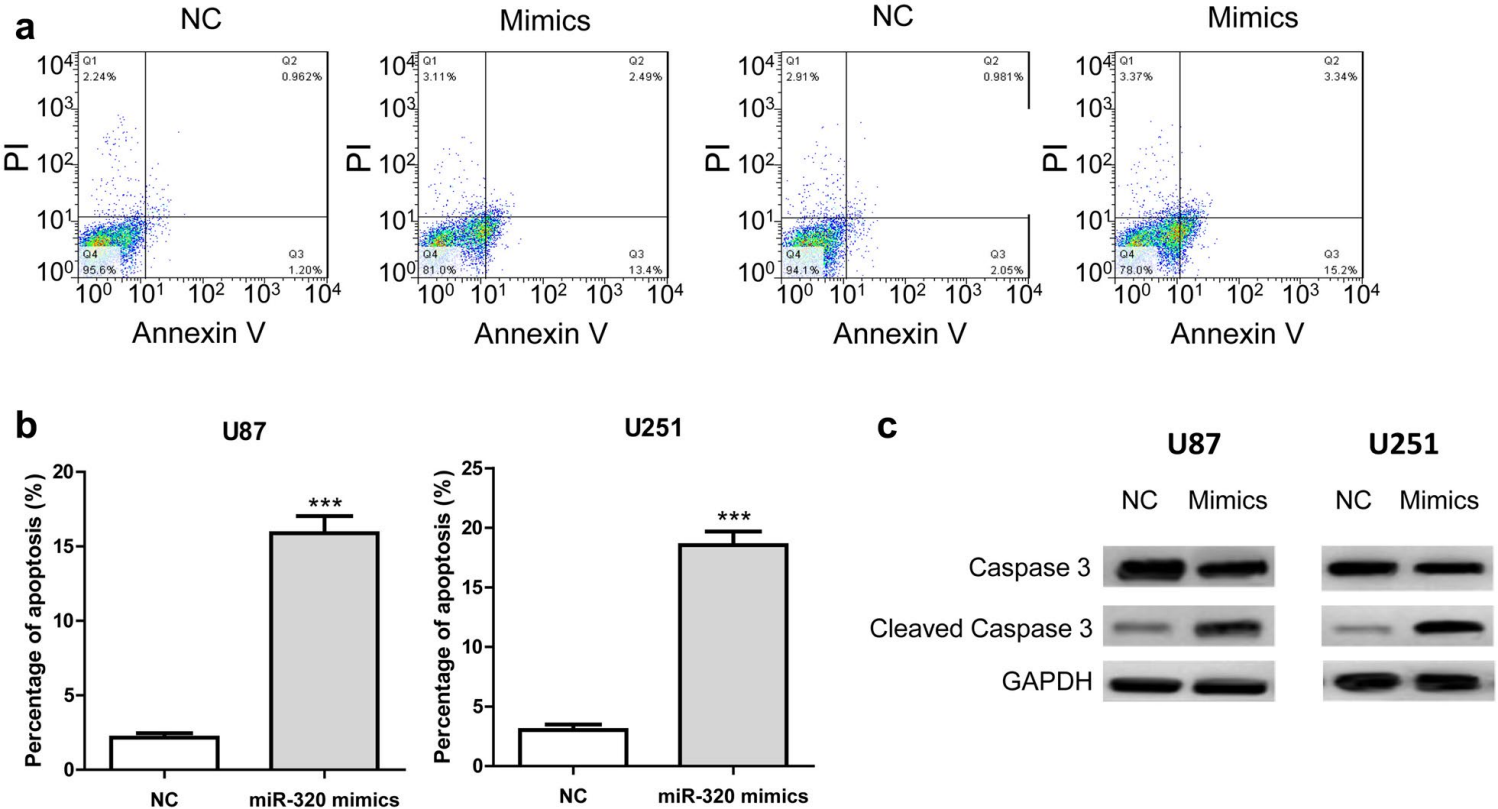
MiR-320 mimics induces apoptosis in U87 and U251 cells. **a** Annexin V/PI dual staining for U87 and U251 cells transfected with miR-320 mimics or NC. **b** Quantitative analysis of cell cytometry results expressed as percentage of the total number of cells counted. **c** A representative result of western blot analysis for caspase-3 protein expression in glioma cells transfected with miR-320 mimics or NC. ***P < 0.001, compared with negative control

The effect of miR-320 mimics on gliomas cell apoptosis was examined using Annexin V and PI double staining assay and western blotting analysis of the caspase-3 protein. Cells were harvested at 48 h after transfection and apoptosis was assessed by FCM. Compared with the control group, the proportion of apoptotic cells in the miR-320 mimic-transfected group was significantly higher (Fig. 4a, b). In addition, miR-320 transfection resulted in caspase-3 activation evidenced by the increased protein level of cleaved caspase-3 (Fig. 4c), suggesting miR-320 mediated glioma cell apoptosis is caspase enzyme dependent.

### MiR-320 overexpression or PBX3 knockdown inhibits MAPK pathway activation in glioma cells

**Fig. 5:**
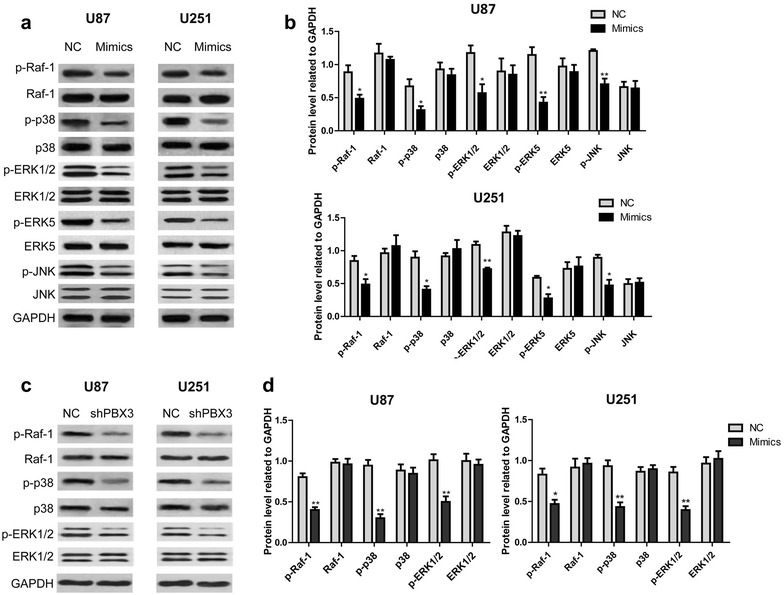
MiR-320 overexpression or PBX3 knockdown inhibits MAPK pathway activation in glioma cells. **a** and **c** After transfection of miR-320 mimics or shPBX3, the activation of Raf-1, p38, ERK1/2, ERK5 and JNK was determined using western blotting assay. **b** and **d** Statistical results of western blot analysis of three independent experiments. *P < 0.05, **P < 0.01, ***P < 0.001, compared with negative control

Several studies have shown that Raf-1/ERK pathway functions as a switch determining cell fate, including proliferation, differentiation, apoptosis, survival and oncogenic transformation [7], [18–20]. In our research, miR-329 mimics is found to significantly decrease the phosphorylation levels of Raf-1, p38, ERK1/2, ERK5 and JNK in both U87 and U251 cells (Fig. 5a, b). To address whether miR-320 functions through targeting PBX3, PBX3 knockdown was performed using shPBX3 and the effect of which on the activation of Raf-1, p38 and ERK1/2 was detected. As shown in Fig. 5c, d, PBX3 knockdown significantly reduced the increased phosphorylation level of Raf-1, p38 and ERK1/2. Taken together, the results presented indicated miR-320 may suppress glioma cell growth through targeting PBX3 and regulating MAPK pathway.

## Discussion

Numerous studies have focused the role of miR-320 in tumor pathogenesis and progress. Wu et al. found miR-320 suppressed tumor angiogenesis driven by vascular endothelial cells in oral cancer by silencing neuropilin 1 [21]. In addition, miR-320 was demonstrated to inhibit osteosarcoma cell proliferation by directly targeting fatty acid synthase [22]. In this study, we found that miR-320 expression was downregulated in glioma tissues. Overexpression of miR-320 suppressed glioma cell proliferation, induced cell cycle arrest and apoptosis. With former relevant researches, the present study suggested that miR-320 may acts as a tumor suppressor.

We identified PBX3 was regulated by miR-320 in glioma cells. Overexpression of PBX3 has been associated with many kinds of malignancies, including gastric cancer [16], colorectal cancer [23], prostate [24] and leukemic [14]. Han et al. found PBX3 is targeted by multiple miRNAs, and is sufficient and necessary for the acquisition and maintenance of tumour-initiating cells (TIC) properties [25]. In the same study, they demonstrated PBX3 drives an essential transcriptional programme, activating the expression of genes critical for hepatocellular carcinoma (HCC) TIC stemness including CACNA2D1, EpCAM, SOX2 and NOTCH3 and the expression of CACNA2D1 and PBX3 mRNA is predictive of poor prognosis for HCC patients [25]. In this research, we found that PBX3 was overexpressed in glioma tissues and was regulated by miR-320, suggested PBX3 may participate in the glioma inhibition function of miR-320. Han et al. found PBX3 was upregulated in colorectal cancer tissues, and over-expression of PBX3 promoted tumour metastasis, both in vitro and in vivo [26]. Li et al. found PBX3 was overexpressed in gastric cancer specimens and cell lines, and positively correlated with disease severity and tumor cell proliferation and invasion [16]. In addition, Han et al. demonstrated high level of PBX3 expression was correlated with the invasive potential of colorectal cancer cells, and significantly associated with lymph node invasion, distant metastasis, advanced TNM stage and poor overall survival of patients [17]. They also found ectopic expression of PBX3 in low metastatic cells was shown to promote migration and invasion [17]. Taken together, PBX3 may be a clinically relevant oncoprotein and a promising therapeutic target of these cancers.

However, the molecular mechanisms responsible for the tumor promoting effect of PBX3 are largely unknown. Han et al. found upregulation of phosphorylated extracellular signal-regulated kinase (ERK)1/2 was one of the targeted molecules responsible for PBX3-induced colorectal cancer cell migration and invasion [17]. Hence, we speculated that PBX3 might also promote glioma by activating MAPK/ERT pathway. Our results showed that either miR-320 mimics transfection or PBX3 knockdown significantly reduced the phosphorylation levels of Raf-1, p38, ERK1/2, ERK5 and JNK were in U87 and U251 cells. The findings suggested miR-320 and PBX3 modulated MAPK pathway may contribute to their effect on the proliferation and apoptosis of glioma cells. The activation of Raf-1 initiates a MAPK cascade that comprises a sequential phosphorylation of the dual-specific MAPK kinases (MAP2K1/MEK1 and MAP2K2/MEK2) and the extracellular signal-regulated kinases (MAPK3/ERK1 and MAPK1/ERK2). The cascade can promote NF-κB activation and inhibit signal transducers involved in motility (ROCK2), apoptosis (MAP3K5/ASK1 and STK3/MST2), proliferation and angiogenesis (RB1). In addition, Raf-1 can also protect cells from apoptosis by translocating to the mitochondria where it binds Bcl-2 and displaces BAD ^29^. PBX3 knockdown significantly suppressed Raf-1 phosphorylation, suggesting PBX3 might aggravate glioma through promoting the activation of Raf-1, and subsequent Raf-1 mediated MAPK cascade and apoptosis inhibition.

In summary, our current data demonstrated that miR-320 is downregulated in glioma tissues and inversely correlates with PBX3 expression. Over expression of miR-320 inhibited glioma cell proliferation and induced cycle arrest and apoptosis. PBX3 was negatively regulated by miR-320 in glioma cells. Either miR-320 overexpression or PBX3 knockdown suppressed the phosphorylation of Raf-1, p38, ERK1/2, ERK5 and JNK. The results suggested miR-320 might functions through the PBX3/Raf-1/MAPK axis, and miR-320 oligonucleotides might be a potential cancer therapeutic for glioma.

## Abbreviations

PBX3: pre-B cell leukaemia transcription factor 3
MTT: 3-(4,5-dimethylthiazol-2-yl)-2,5-diphenyl tetrazolium bromide
GBM: glioblastoma multiforme
3′-UTRs: 3′-untranslated regions
qRTPCR: real-time quantitative PCR
PBS: phosphate-buffered saline
MAPK: mitogen-activated protein kinase

## References

[1] Holland EC (2000). Glioblastoma multiforme: the terminator. Proc Natl Acad Sci USA.

[2] Stupp R, Mason WP, van den Bent MJ (2005). Radiotherapy plus concomitant and adjuvant temozolomide for glioblastoma. New Engl J Med.

[3] Van Meir EG, Hadjipanayis CG, Norden AD, Shu HK, Wen PY, Olson JJ (2010). Exciting new advances in neuro-oncology: the avenue to a cure for malignant glioma. CA Cancer J Clin.

[4] Ameres SL, Zamore PD (2013). Diversifying microRNA sequence and function. Nat rev. Mol cell biol.

[5] Sun K, Lai EC (2013). Adult-specific functions of animal microRNAs. Nat rev. Genet.

[6] Kent OA, Mendell JT (2006). A small piece in the cancer puzzle: microRNAs as tumor suppressors and oncogenes. Oncogene.

[7] Esquela-Kerscher A, Slack FJ (2006). Oncomirs—microRNAs with a role in cancer. Nat rev. Cancer.

[8] Tadano T, Kakuta Y, Hamada S (2016). MicroRNA-320 family is downregulated in colorectal adenoma and affects tumor proliferation by targeting CDK6. World J Gastrointest Oncol.

[9] Engelmann D, Putzer BM (2012). The dark side of E2F1: in transit beyond apoptosis. Can Res.

[10] Yao J, Liang LH, Zhang Y (2012). GNAI1 Suppresses Tumor Cell Migration and Invasion and is Post-Transcriptionally Regulated by Mir-320a/c/d in Hepatocellular Carcinoma. Cancer biol med.

[11] Hsieh IS, Chang KC, Tsai YT (2013). MicroRNA-320 suppresses the stem cell-like characteristics of prostate cancer cells by downregulating the Wnt/beta-catenin signaling pathway. Carcinogenesis.

[12] Bronisz A, Godlewski J, Wallace JA (2011). Reprogramming of the tumour microenvironment by stromal PTEN-regulated miR-320. Nat Cell Biol.

[13] Dong L, Li Y, Han C, Wang X, She L, Zhang H (2014). miRNA microarray reveals specific expression in the peripheral blood of glioblastoma patients. Int J Oncol.

[14] Li Z, Zhang Z, Li Y (2013). PBX3 is an important cofactor of HOXA9 in leukemogenesis. Blood.

[15] Dickson GJ, Liberante FG, Kettyle LM (2013). HOXA/PBX3 knockdown impairs growth and sensitizes cytogenetically normal acute myeloid leukemia cells to chemotherapy. Haematologica.

[16] Li Y, Sun Z, Zhu Z, Zhang J, Sun X, Xu H (2014). PBX3 is overexpressed in gastric cancer and regulates cell proliferation. Tumour Biol J Int Soc Oncodev Biol Med.

[17] Han HB, Ji DB, Li ZW (2014). PBX3 promotes migration and invasion of colorectal cancer cells via activation of MAPK/ERK signaling pathway. World J Gastroenterol.

[18] Dubois T, Rommel C, Howell S (1997). 14-3-3 is phosphorylated by casein kinase I on residue 233. Phosphorylation at this site in vivo regulates Raf/14-3-3 interaction. J Biol chem.

[19] von Kriegsheim A, Pitt A, Grindlay GJ, Kolch W, Dhillon AS (2006). Regulation of the Raf-MEK-ERK pathway by protein phosphatase 5. Nat Cell Biol.

[20] Chen J, Fujii K, Zhang L, Roberts T, Fu H (2001). Raf-1 promotes cell survival by antagonizing apoptosis signal-regulating kinase 1 through a MEK-ERK independent mechanism. Proc Natl Acad Sci USA.

[21] Wu YY, Chen YL, Jao YC, Hsieh IS, Chang KC, Hong TM (2014). miR-320 regulates tumor angiogenesis driven by vascular endothelial cells in oral cancer by silencing neuropilin 1. Angiogenesis.

[22] Cheng C, Chen ZQ, Shi XT (2014). MicroRNA-320 inhibits osteosarcoma cells proliferation by directly targeting fatty acid synthase. Tumour Biol J Int Soc Oncodev Biol Med.

[23] Han HB, Gu J, Ji DB (2014). PBX3 promotes migration and invasion of colorectal cancer cells via activation of MAPK/ERK signaling pathway. World J Gastroenterol.

[24] Ramberg H, Grytli HH, Nygard S (2016). PBX3 is a putative biomarker of aggressive prostate cancer. Int J Cancer.

[25] Han H, Du Y, Zhao W (2015). PBX3 is targeted by multiple miRNAs and is essential for liver tumour-initiating cells. Nat commun.

[26] Han HB, Gu J, Zuo HJ (2012). Let-7c functions as a metastasis suppressor by targeting MMP11 and PBX3 in colorectal cancer. J Pathol.

